# Association between RANTES Gene Polymorphisms and Asthma: A Meta-Analysis

**DOI:** 10.1371/journal.pone.0090460

**Published:** 2014-06-25

**Authors:** Dan Wen, Xin Du, Shao-Ping Nie, Jian-Zeng Dong, Chang-Sheng Ma

**Affiliations:** Department of Cardiology, Beijing Anzhen Hospital, Capital Medical University, Beijing, China; University of Leuven, Rega Institute, Belgium

## Abstract

**Background:**

A few recent studies have suggested that regulated on activation, normal T cell expressed and secreted (RANTES) polymorphisms (−403 G/A, −28C/G) are associated with asthma. However, there still existed studies which did not confirm these correlations.

**Objective:**

The objective of this study was to evaluate the relationship of RANTES and asthma using a meta-analysis.

**Methods:**

Pubmed, Embase, and Cochrane library databases were systemically searched. Data were extracted by two independent reviewers and pooled odds ratio (OR) with 95% confidence interval (CI) were calculated.

**Results:**

Eighteen studies were enrolled, including a total of 2558 cases and 2630 controls of −403 G/A, as well as 3311 cases and 4031 controls of −28C/G in this meta-analysis. The overall ORs and 95% CIs of −403 G/A were 1.19, 1.06–1.33 (P<0.001) and 1.25, 1.03–1.51 (P = 0.933) in dominant and recessive models, respectively. The overall ORs and 95% CIs of −28G were 1.23, 1.09–1.39 (P = 0.221) and 1.76, 1.32–2.34 (P = 0.356) in dominant and recessive models, respectively. No publication bias among studies was showed.

**Conclusions:**

This meta-analysis showed that RANTES −403 G/A polymorphism was a risk factor for asthma, while −28C/G polymorphism were not associated with asthma.

## Introduction

Asthma is a common chronic respiratory inflammation disease associated with airway hyperreactivity, reversible airway obstruction, mucus hypersecretion, inflammatory cell migration, bronchial epithelial desquamation, and airway wall remodeling [1], [2]. It is a complex disorder caused by both genetic and environmental factors. Over 100 genes have been reported to be associated with asthma risk and related phenotypes [3], [4].

The regulated on activation, normal T cell expressed and secreted (RANTES), a member of the CC chemokine family, is a potent eosinophil, monocyte, basophile and lymphocyte chemo-attractant. It had close correlations with the attraction and recruitment of lymphocytes, monocytes, basophils and eosinophils to the places of inflammation, and thus, was involved in various inflammatory and immune disorders, including asthma [5], [6], [7]. Two RANTES promoter polymorphisms of −403 G/A and −28C/G were demonstrated to affect the promoter activity and increase the expression of RANTES [8].

Although numerous studies have demonstrated the correlation between RANTES (−403G/G and −28C/C) polymorphisms and asthma, the results still remains inconsistent. Therefore, this meta-analysis was performed currently to observe the association of these two polymorphisms with asthma risk, which firstly provided the updated meta-analysis of comprehensive studies about RANTES gene polymorphisms and asthma.

## Methods

### 2.1 Search strategy

Two independent reviewers searched Pubmed, Embase, and Cochrane library databases systemically and extensively to obtain the case-control genetic association of RANTES polymorphisms and asthma studies without any language restrictions. The Medical Subject Heading (MeSH) and keyword terms “RANTES”, “CCL5”, “asthma”, and “polymorphism” were used as search criteria.

### 2.2 Study selection and data abstraction

The inclusion criteria for the gene association studies in this meta-analysis were as follows: 1) case-control studies and cohort studies; 2) original data on genotype and allele distributions and frequencies were available for case and control subjects; 3) genotype distributions of the controls were in Hardy-Weinberg equilibrium. Data abstraction was performed by two independent reviewers as mentioned above.

### 2.3 Statistical analysis

Chi-square test was used to determine whether the genotype distributions of the controls were in Hardy-Weinberg equilibrium. Heterogeneity between studies was tested with both Cochran's test and *I^2^* statistics. P<0.1 or *I^2^*>50% indicated significant heterogeneity in this study [9]. Publication bias was assessed by funnel plot and Egger's regression test [10]. Data of this meta-analysis were analyzed by Stata software (Version 12.0; Stata Corporation, College Station, TX). P-value <0.05 were considered statistically significant.

## Results

By searching Pubmed, Embase, and Cochrane library databases systemically and extensively, one was excluded because of unavailable data [11]. A total of 18 case-control and cohort studies with usable data met the inclusion criteria and were enrolled in this meta-analysis, including 14 articles on −403 G/A, and 14 on −28C/G [12]–[29]. The included studies provided 2558 cases and 2630 controls of −403 G/A, as well as 3311 cases and 4031 controls of −28C/G for this analysis. The characteristics of included studies in this present meta-analysis were showed in Table 1 and Table 2.

**Table 1:** Characteristics of included studies in the meta-analysis for the −403G/A polymorphism.

First Author (Ref)	Year	Sample size (case/control)	Case genotypes	Control genotypes
Fryer (19)	2000	120/74	75/39/6	51/21/2
Szalai (12)	2001	164/303	122/32/6	211/84/8
Hizawa (13)	2002	298/311	146/108/44	140/137/34
Yao (14)	2003	182/107	98/65/19	60/41/6
Liu (21)	2005	32/32	17/13/2	16/14/2
Leung (17)	2005	129/66	60/53/16	37/21/8
Moissidis (18)	2005	61/131	16/34/11	35/72/24
Al-Abdulhadi (16)	2005	162/291	47/98/17	166/104/21
Lachheb (23)	2007	210/224	140/50/20	174/40/10
Sohn (24)	2008	326/253	109/146/71	97/107/49
Tölgyesi G (22)	2006	144/174	107/34/3	131/40/3
Muro (25)	2008	306/242	202/93/11	165/69/8
Nahas (27)	2012	40/38	30/10/0	30/8/0
Liu (27)	2013	384/384	148/186/50	149/183/52

**Table 2:** Characteristics of included studies in the meta-analysis for the −28C/G Polymorphism.

First Author (Ref)	Year	Sample size (case/control)	Case genotypes	Control genotypes
Szalai (12)	2001	164/303	144/16/0	284/19/0
Hizawa (13)	2002	298/311	216/70/12	243/62/6
Yao (14)	2003	182/107	134/39/9	83/23/1
Wang (15)	2004	100/90	65/31/4	72/17/1
Huang (20)	2005	251/107	189/53/9	83/23/1
Moissidis (18)	2005	61/129	59/2/0	129/0/0
Liu (21)	2005	32/32	25/6/1	29/3/0
Lachheb (23)	2007	210/224	163/35/12	190/29/5
Muro (25)	2008	306/242	289/17/0	228/14/0
Sohn (24)	2008	326/253	218/93/15	174/66/13
Murk (26)	2011	100/482	1/1/98	0/25/457
Nahas (27)	2012	40/38	40/0/0	37/1/0
Liu (28)	2013	384/384	282/63/39	310/60/14
Kaneko (29)	2013	857/1329	625/210/22	984/310/35

**Figure 1:**
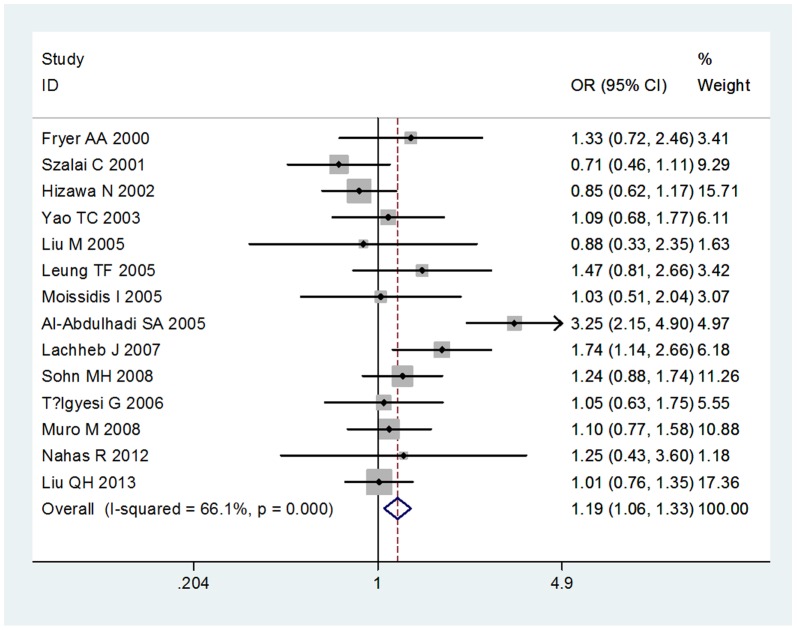
The association between RANTES-403G/A polymorphism and asthma in dominant model.

**Figure 2:**
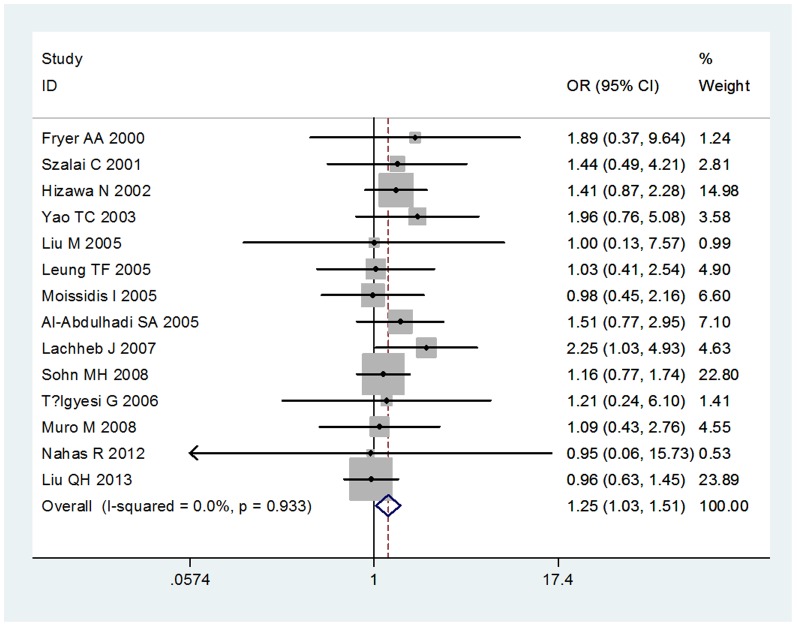
The association between RANTES-403G/A polymorphism and asthma in recessive model.

**Figure 3:**
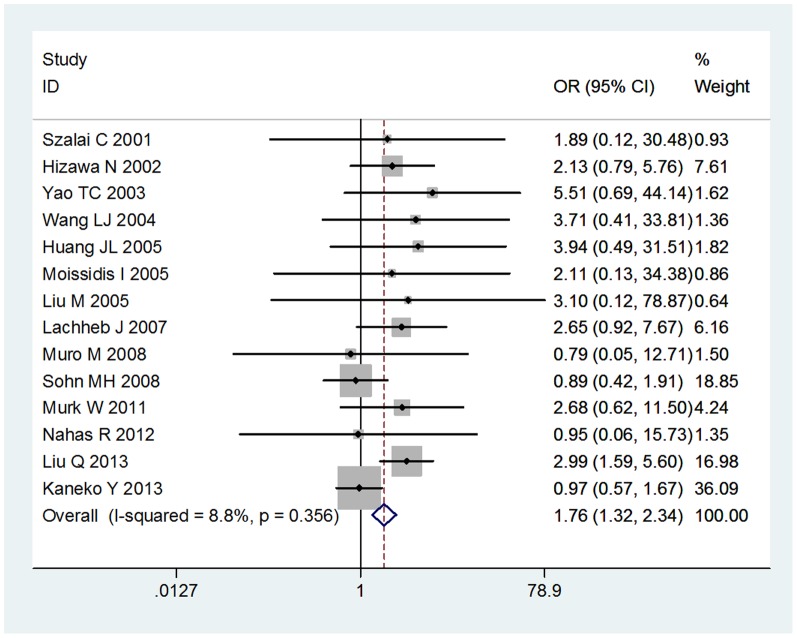
The association between RANTES −28C/G polymorphism and asthma in dominant model.

**Figure 4:**
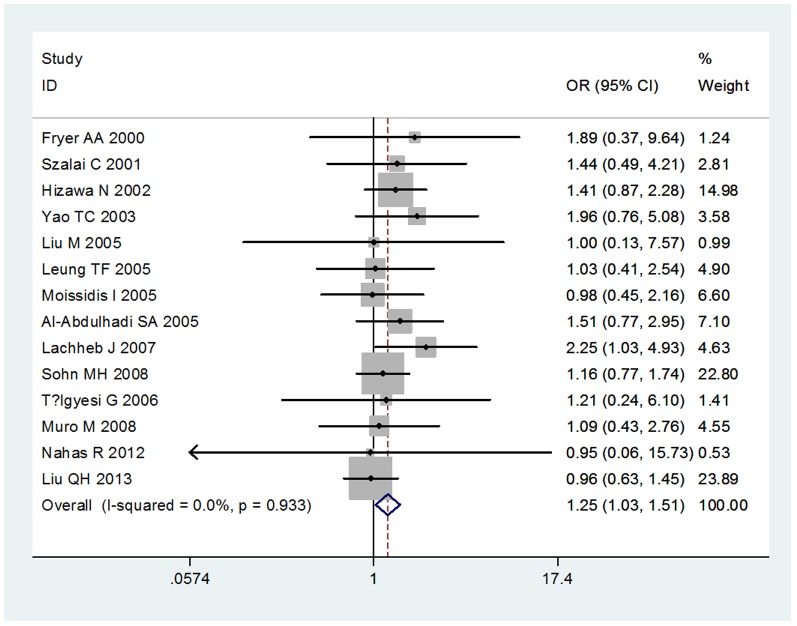
The association between RANTES −28C/G polymorphism and asthma in recessive model.

We compared the minor allele to major allele in dominant, recessive, and additive models. The overall ORs and 95% CIs of −403 G/A were 1.19, 1.06–1.33 (P<0.001) and 1.25, 1.03–1.51 (P = 0.933) in dominant and recessive models, respectively (Figure 1,2). The overall ORs and 95% CIs of −28G were 1.23, 1.09–1.39 (P = 0.221) and 1.76, 1.32–2.34 (P = 0.356) in dominant and recessive models, respectively (Figure 3,4) (Table 3). Funnel plot and Egger's regression test showed no publication bias among studies of −403 G/A (P = 0.743 and 0.400 in dominant and recessive, respectively) and −28C/G (P = 0.435 and 0.244 in dominant and recessive, respectively).

**Table 3:** The association between RANTES gene polymorphisms and asthma in different genetic models.

Gene polymorphism	Number of studies	Genetic model	OR	95% CI	P value
−403G/A	14	Dominant	1.19	1.06–1.33	<0.001
		Recessive	1.25	1.03–1.51	0.933
−28C/G	14	Dominant	1.23	1.09–1.39	0.221
		Recessive	1.76	1.32–2.34	0.356

OR: odds ratio; CI: confidence interval.

## Discussion

Data of this meta-analysis showed that RANTES −403 G/A polymorphism was a risk factor for asthma, while −28C/G polymorphism were not associated with asthma.

RANTES, also known as C-C motif chemokine ligand 5 (CCL5), is a potent chemoattractant which play an important role in immune and inflammatory regulation process. The RANTES gene is located on chromosome 17q11.2-q12, which is composed of three exons and two introns. It was reported that RANTES gene polymorphisms could influence the activity of transcription and protein expression in human [30]. Significantly elevated concentrations of RANTES were observed in asthmatic patients, and associated with asthma severity, demonstrating the important role of RANTES in the pathogenesis of this disorder [31], [32], [33]. Serum RANTES may a helpful noninvasive and diagnostic marker for monitoring asthma severity. Identification and blocking of RANTES and/or its receptor may be a promising therapeutic approach to asthmatic patients [34]. Previous investigations have reported the association of RANTES gene polymorphisms (−403G/A and −28C/G) and asthma susceptibility, however, findings of the possible relationships are remain inconsistent.

In Chinese asthmatic children population, Leung et al. [17] found that RANTES −403G/A polymorphism was associated with allergen sensitization and forced expiratory volume in 1-s (FEV_1_), and no relation was observed in −28C/G polymorphism. On the contrary, numerous observations [14], [20], [28] reported opposite conclusions showing that −28C/G polymorphism may exacerbate asthma severity. In contrast to these results, neither −403G/A nor −28C/G was indicated to be associated with asthma [11], [21].

In other countries, both −403G/A and −28C/G polymorphisms did not have a detectable effect on asthma susceptibility in African Americans, Lebanon, Spanish or Budapest population, respectively [12], [18], [25], [27]. However, Lachheb et al. [23] suggested that both polymorphisms may play an important role in asthma predisposition, airway obstruction severity, or bronchial hyperresponsiveness among Tunisian or Korean children. Moreover, several investigations also demonstrated either −403G/A or −28C/G was related to asthma risk [16], [19], [29].

In this present study, we found that RANTES −403 G/A polymorphism was a risk factor for asthma susceptibility under dominant genetic model, indicating its potential role in asthma pathogenesis. In the meanwhile, the data also indicated that −28C/G polymorphism was not associated with asthma risk. Furthermore, no publication bias among studies was showed. Up to now, there were four meta-analysis investigations indicating controversial results about the correlation between these two polymorphisms and asthma. Three studies about the association of RANTES gene polymorphisms and asthma susceptibility reported that −28C/G polymorphism could increase the risk of asthma in Asian children or pediatric asthma in global population, while no relationship was found in −403G/A [35], [36], [37]. However, Zhang et al. [38] observed contrary findings showing that −403G/A polymorphism would be a risk factor among atopic asthma patients, and no such association was indicated in −28C/G, which is consistent with our findings. Compared with previous studies, our present study firstly provided the updated meta-analysis of comprehensive studies about RANTES gene polymorphisms and asthma.

The susceptibility of asthma might be due to the interactions of various genes (including linkage among gene polymorphisms), environment and ethnic heterogeneity factors. Therefore, larger scale studies are required to provide confirm evidence on the roles of RANTES (−403A/G and −28C/G) polymorphisms in asthma risk.

## Conclusions

In summary, we concluded that RANTES −403 G/A polymorphism was a risk factor for asthma, while −28C/G polymorphism were not associated with asthma.

## Supporting Information

Checklist S1 **PRISMA Checklist.**
(DOC)
