# Correction: Association between RANTES Gene Polymorphisms and Asthma: A Meta-Analysis

**DOI:** 10.1371/journal.pone.0111818

**Published:** 2014-10-17

**Authors:** The PLOS ONE Staff

Figures 3 and 4 are incorrect. The authors have provided corrected versions here.

**Figure 3:**
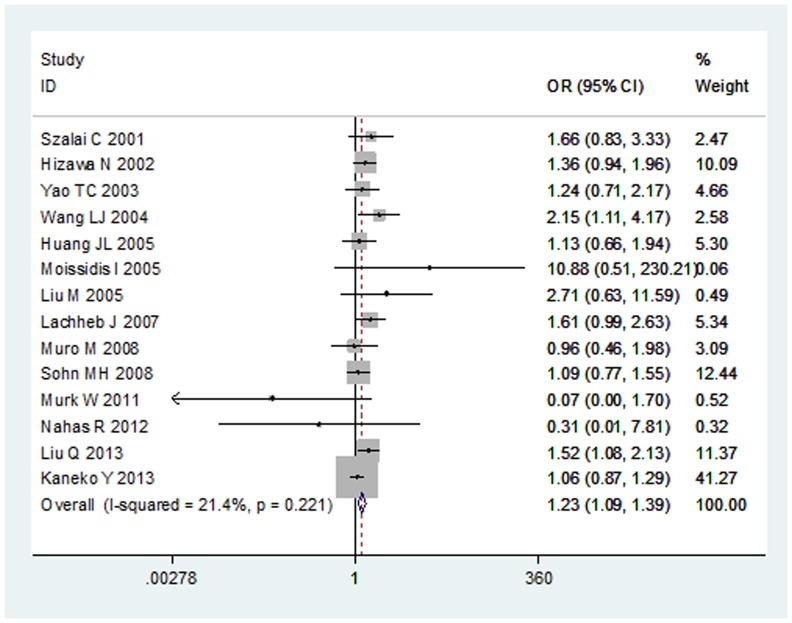
The association between RANTES −28C/G polymorphism and asthma in dominant model.

**Figure 4:**
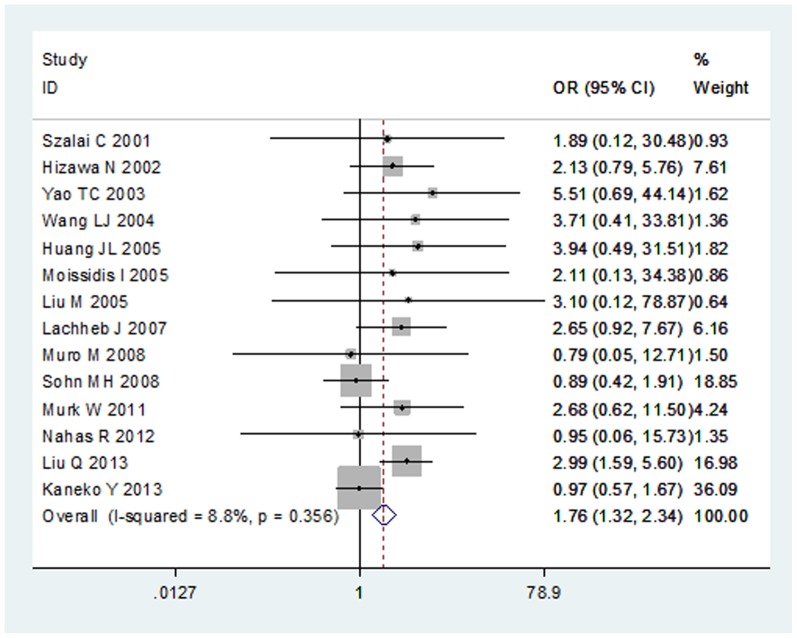
The association between RANTES −28C/G polymorphism and asthma in recessive model.

In addition, some information is missing from the Funding section. The correct Funding statement is: The study was supported by grants from Beijing Municipal Administration of Hospitals Clinical Medicine Development of Special Funding Support (ZY201302), Ministry of S&T Program of Chinese Arrhythmia Registration Study (2013BAI09B02), and International S&T Cooperation Program of China (No. 2013DFB30310). The funders had no role in study design, data collection and analysis, decision to publish, or preparation of the manuscript.
